# Serum 25-hydroxyvitamin D3 levels and vitamin D receptor variants in melanoma patients from the Mediterranean area of Barcelona

**DOI:** 10.1186/1471-2350-14-26

**Published:** 2013-02-16

**Authors:** Zighereda Ogbah, Laura Visa, Celia Badenas, José Ríos, Joan Anton Puig-Butille, Nuria Bonifaci, Elisabet Guino, Josep Maria Augé, Isabel Kolm, Cristina Carrera, Miquel Ángel Pujana, Josep Malvehy, Susana Puig

**Affiliations:** 1 Melanoma Unit, Department of Dermatology Hospital Clínic de Barcelona, IDIBAPS, Barcelona University, Barcelona, Spain; 2 Melanoma Unit, Biochemistry and Molecular Genetics Service, Hospital Clínic de Barcelona, IDIBAPS, Barcelona University, Barcelona, Spain; 3 Centre of Biomedical Research on Rare Diseases (CIBERER), ISCIII, Barcelona, Spain; 4 Statistics and Methodological Support Unit, Unitat d’Avaluació, Suport i Prevenció (UASP), Hospital Clínic, IDIBAPS, Barcelona, Spain; 5 Bioinformatics and Biostatistics Unit, and Translational Research Laboratory, Catalan Institute of Oncology, Bellvitge Biomedical Research Institute (IDIBELL), L’Hospitalet, Barcelona, Spain

**Keywords:** Vitamin D, *VDR*, SNP, Melanoma

## Abstract

**Background:**

Serum 25-hydroxyvitamin D3 (Vitamin D) insufficiency and single-nucleotide polymorphisms (SNPs) on its receptor, Vitamin D receptor (*VDR*), have been reported to be involved in melanoma susceptibility in populations mostly from northern countries.

**Objective:**

To investigate 25-hydroxyvitamin D3 levels and *VDR* SNPs in melanoma patients from sunny area of Barcelona, two studies were carried out. The first study evaluated the levels of Vitamin D at time of melanoma diagnosis and the second one analyzed the association between *VDR* genetic variants and risk of having a high nevus number, the strongest phenotypic risk factor for melanoma.

**Methods:**

The levels of 25-hydroxyvitamin D3 in 81 melanoma patients at diagnosis were measured. In a second group of melanoma patients, including 150 with low and 113 with high nevus number, 11 *VDR* SNPs were analyzed for their association with nevus number.

**Results:**

In the first study, 68% of patients had insufficient levels of 25-hydroxyvitamin D3 (<25 ng/ml). Autumn-winter months and fair phototype were associated with 25-hydroxyvitamin D3 insufficiency; after multivariate analysis, season of sampling remained the only independent predictor of 25-hydroxyvitamin D3 levels. In the second study, *VDR* variant rs2189480 (*P* = 0.006) was associated with risk of high nevus number whereas rs2239179 (*P* = 0.044) and rs7975128 (*P* = 0.0005) were protective against high nevus number. After Bonferroni adjustment only rs7975128 remained significant. In stratified analysis, SNP rs7975128 was found protective against multiple melanomas (*P* = 0.021).

**Conclusion:**

This study showed that even in Barcelona, a sunny Mediterranean area, 25-hydroxyvitamin D3 levels were sub-optimal in the majority of melanoma patients at diagnosis. The involvement of VDR in nevi and, in turn, in melanoma susceptibility has also been suggested. Larger studies are needed to confirm our findings.

## Background

Serum 25-hydroxyvitamin D3 (Vitamin D) is a hormone classically known for its regulation of bone and calcium metabolism. In recent years interest in 25-hydroxyvitamin D3 has grown as evidence has shown it to play a role in cancer susceptibility and outcome, postulated to be related to its anti-proliferative pro-apoptotic and pro-differentiation properties [1,2]. Suboptimal levels of 25-hydroxyvitamin D3 have been shown to be associated with increased risk of several cancers including melanoma [3,4]. Patients diagnosed with melanoma had suboptimal levels of 25-hydroxyvitamin D3 in the North of England within a few months of diagnosis, especially in winter months [5] and a reduced level of 25-hydroxyvitamin D3 was associated with thicker melanomas, poorer outcome [5] and aggressive melanoma [6]. There are no reports of 25-hydroxyvitamin D3 levels in melanoma patients living in sunny Mediterranean countries and we therefore herein report a small study in melanoma patients from Barcelona.

Sunburn and intermittent sun exposure are the most important environmental risk factors for melanoma [7] but sun exposure is also the main source for 25-hydroxyvitamin D3 photosynthesis. 25-hydroxyvitamin D3 derives from cholesterol metabolites in skin after exposure to ultraviolet B radiation (UVB) [8]. The skin exposure to UVB induces the photolytic conversion of 7-dehydrocholesterol (7-DHC) to previtamin D and from there to vitamin D3. The metabolic activation of vitamin D3 requires two hydroxylation steps [9]. The first hydroxylation occurs in the liver where vitamin D3 is converted in 25-hydroxyvitamin D3, the major circulating metabolite of vitamin D_3_. The second hydroxylation transformed in the kidney the 25-hydroxyvitamin D3 in 1,25-dihydroxyvitamin D3. While circulating 1,25-dihydroxyvitamin D3 levels mainly reflect the synthesis of 1,25-dihydroxyvitamin D3 in the kidney, 25-hydroxyvitamin D3 serum levels are a good parameter of whole body vitamin D status and for this reason is the metabolite most measured for the classification of vitamin D status [10]. In vitro, 25-hydroxyvitamin D3 has been shown to inhibit the growth of normal melanocytes and melanocytes derived from melanoma [11,12]. The 25-hydroxyvitamin D3 mediates its biological effects by binding to its specific nuclear receptor, Vitamin D Receptor (*VDR)*[13]*.*

The *VDR* gene, located on chromosome 12q13.11, has more than 200 single-nucleotide polymorphisms (SNPs) [14] but only a few SNPs, considered to be functional [15], have been studied for their potential associations with melanoma susceptibility [16-21]. Meta-analysis of all reports published by 2009 supported the association between *VDR* functional SNPs *Bsm*I (rs1544410) and *Fok*I (rs2228570) and melanoma risk [22,23]. More recently a large SNP study showed that common inherited variants at *VDR* influence melanoma risk [24], providing further evidence supporting a role for 25-hydroxyvitamin D3 in melanoma susceptibility.

Common acquired nevi are melanocytes in proliferation and high nevus number represents the strongest phenotypic risk factor for melanoma development [25,26]. Genome-wide association studies have identified common susceptibility genes for melanoma and increased nevus number [27,28].

If *VDR* SNPs are associated with melanoma risk therefore, elucidating the relationship between *VDR* variants and nevi is an important step towards understanding the involvement of *VDR* and, indirectly, of 25-hydroxyvitamin D3, in melanoma carcinogenesis.

The association between 25-hydroxyvitamin D3 levels and/or inherited variation in *VDR* and melanoma risk has been studied in few populations, mostly from northern countries such as England, USA, Poland and Germany [4,6,21-23]. Only two reports on *VDR* variants have been published from southern European countries, specifically Italy and Spain [18,29].

We presented the results of two studies performed in Barcelona melanoma patients. The first study evaluated the levels of 25-hydroxyvitamin D3 at time of melanoma diagnosis and the second one analyzed the association between *VDR* genetic variants and risk of having a high nevus number.

## Methods

### Study design

Two cohort studies both including Caucasian melanoma patients were carried out. The two studies were mutually exclusive (each patient is present in only one study) and included groups of patients belonging to two different studies. In the first, a retrospective cohort study, 25-hydroxyvitamin D3 levels were measured in serum samples stored from melanoma patients at diagnosis for the investigation of biomarkers. In the second, the association between *VDR* variants and nevus number was evaluated in a second cohort of melanoma patients recruited to a genetic epidemiological study of nevi. Patients were selected according to the total nevus number at the time of melanoma diagnosis and classified into two groups: 150 cases with a low nevus number (<50 nevi) and 113 cases with a high nevus number (>100 nevi). We selected two groups of patients where there would be no possible overlap between them, as <50 nevi were closer to the normal nevus number in our population [30] and >100 nevi being a well- documented risk factor for melanoma [26].

Both cohort studies included melanoma patients from the urban area of Barcelona recruited from the Melanoma Unit of Hospital Clinic of Barcelona. This is a referral centre for Melanoma in the Catalonia region.

Both studies were approved by the Ethics Committee of the Hospital Clinic of Barcelona and written informed consent was obtained from all participants in the study.

### The first set of melanoma patients (25-hydroxyvitamin D3 study)

The patients had all been diagnosed with melanoma between 2004–2008. Patients were those recruited to the epidemiological study from which serum samples had been cryopreserved in the Biobank of Hospital Clínic and where the blood was collected within a maximum of 3 months after melanoma diagnosis. All patients had localized tumors (stage I and stage II) except one (stage IIIa) which had lymph node micrometastasis. Data on gender, age at melanoma diagnosis, nevus number, Fitzpatrick skin type, eye color, hair color, examined actinic damage, number of melanoma, Breslow thickness, reported solar exposure before 10 years of age, between 10–18 and over 18 years were collected through a questionnaire or extracted by medical records. Participants were asked about weight, height and habits of photoprotection via an interview.

### The second set of melanoma patients (*VDR* polymorphisms study)

A total of 263 melanoma patients were recruited between 2004–2007. The patients were selected according to the total number of nevi at the time of melanoma diagnosis and classified into two groups: 150 cases with a low nevus number (cases with <50) and 113 cases with a high nevus number (cases with >100). Total body nevus number has been performed by dermatologists and trained nurses. Data on gender, age at melanoma diagnosis, nevus number, Fitzpatrick skin type, eye color, hair color, examined actinic damage, number of melanoma, reported solar exposure as before 10 years of age, between 10–18 and over 18 years were collected through a questionnaire or extracted by medical records.

### 25-hydroxyvitamin D3 measurement

The levels of 25-hydroxyvitamin D3 (ng/ml) were analyzed by a chemiluminescence immunoassay method using the Liaison ® kit (DiaSorin, Minnesota, USA) as previously described [31]. This method has a detection limit range from 4 ng/ml to 150 ng/ml. Insufficient level of 25-hydroxyvitamin D3 was defined as less than 25 ng/ml (60 nmol/L).

### *VDR* polymorphisms genotyping

In total, 11 *VDR* SNPs rs7136534, rs11574027, 11168287, rs2238136, rs3782905, rs2189480, rs2239179, rs11574077, rs11168267 and rs7975128 were analyzed. All SNPs were selected as having minor allele frequencies (MAF) ≥ 0.05 in HapMap (http://www.hapmap.org) European individuals and at least one independent validation criterion, as established in dbSNP (http://htpp://www.ncbi.nlm.nih.gov/snp). The SNPs were selected using data from the HapMap project and the Tagger tool [32] with linkage disequilibrium r^2^ > 0.5.

Genomic DNA was isolated from peripheral blood samples using the salting-out method. Genotyping was performed with GenomeLab SNPstream genotyping platform (Beckman & Coulter Inc. Fullerton, CA) and its accompanying SNPstream software, according to the manufacturer’s protocols. Primers were designed using Autoprimer (http://www.autoprimer.com). The design of the primers failed in the presence of adjacent SNPs or of repeat sequence which can interfere with primers annealing. If a selected SNP failed assay primer design, if possible, an alternative tagging SNP in complete LD was chosen. Two SNPs, *Fok*I (rs2228570) and *Bsm*I (rs1544410), failed primer design. By HapMap, *Bsm*I was replaced with rs7975128 as in strong LD (r2 = 1) in the Caucasian population while no other variants in LD were found for *Fok*I. Samples and SNPs with call rates below 90% were excluded from the analysis. Failed genotypes were not repeated. As quality controls, 10% of samples were added as duplicates so that concordance between genotype calls could be assessed. The genotyping assays were performed at the LIMM (Leeds) genotyping facilities.

### Statistical method

Quantitative variables were described using median and interquartile range (percentiles 25^th^ and 75^th^). Inferential analyses for these variables were performed by U Mann–Whitney or Wilcoxon test for unpaired data. Qualitative variables were tabulated by absolute frequencies and percentages and evaluated using *χ*^2^ test or Fisher’s exact test where appropriate.

### 25-hydroxyvitamin D3

The analysis was performed using SPSS v15.0 (SPSS Inc., Chicago IL). The level of statistical significance was set at *P* < 0.05. Logistic regression was used to estimate Odds Ratio (OR) and 95% Confidence Interval (CI) for association between 25-hydroxyvitamin D3 levels (<25 ng/ml) and gender, age diagnosis of melanoma (<55/>55), BMI (normal weight/overweight/obese), Fitzpatrick skin type (dark/fair), eye color (dark/light), hair color (dark/light), actinic damage (no/yes); habits of photoprotection (usually/never), solar exposure (none to mild, that is under 50 hour per year (h/y) or moderate to severe that is more than 50 (h/y)) before 10 years of age, between 10–18 and over 18 years, number of melanoma (single/multiple) and Breslow (<0.75 mm/0.75-1 mm/>1 mm). For the purpose of the analysis, Breslow thickness was also analyzed as a continuous variable. The lineal trend of association between 25-hydroxyvitamin D3 levels and Breslow thickness was evaluated by Spearman correlation. In this cohort, cases with an intermediate nevus number between 51 and 99 (51–99) were present; nevus number variable included three groups (≤50 nevi), (51–99) and (≥100 nevi).

To test the effect of the season on blood sampling as an independent factor, a multivariate approach was performed adjusting by phototype and gender.

### *VDR* polymorphisms

Statistical analyses were performed using SNPassoc package in R (version 2.10.1) [33]. Departure from Hardy–Weinberg equilibrium (HWE) for each SNP was assessed by *χ*^2^ test. Being a study including only cases, SNPs found outside HWE were analyzed because HWE could be informative for SNPs that confer risk rather than genotyping error. To measure the association between *VDR* variant alleles and risk for increased number of nevi, cases with less than 50 nevi were compared with cases with more than 100 nevi, where cases <50 were considered as the referent group.

The association between genotype and nevus number was estimated using logistic regression and analyzed under co-dominant, dominant, recessive and log-additive models. Significance was defined as *P* < 0.05. The best inheritance model was selected using the Akaike criteria. The reported ORs and 95% CI were adjusted for age and gender. Significant associations between SNPs and nevus number were also tested by the Bonferroni test. To control confounder variables, regression analysis was repeated adjusting for whether there was a single or multiple primaries. Logistic regression was also used to estimate the association between polymorphisms and clinical characteristics such as eye color (dark/light), hair color (dark/light), Fitzpatrick skin phototype (dark/fair), actinic damage (no/yes) and number of melanomas (single/multiple). Associations between *VDR* SNPs and solar exposure (none-mild < 50 h/y/severe-moderate > 50 h/y) before 10, between 10–18 and over 18 years was evaluated by interaction analysis.

## Results

### 25-hydroxyvitamin D3

Medians levels of 25-hydroxyvitamin D3 and the clinical characteristics of patients are shown in Table 1. A total of 81 patients were analyzed, of whom 38 (47%) were female. For women, the median was 22.8 ng/ml and for men, 19.59 ng/ml (*P* = 0.477). The median (25^th^ - 75^th^ percentiles) age at melanoma diagnosis was 55 (36.7- 67.2). It was observed that younger patients (<55 years) had slightly higher levels of 25-hydroxyvitamin D3 (median 22.5 ng/ml) than older patients (>55 years) (median 19.7 ng/ml) (*P* = 0.441).

**Table 1:** Descriptive analysis of characteristics and medians of 25-hydroxyvitamin D3 levels at time of diagnosis in melanoma patients

	**Variables**		Nº patients*	**Vitamin D levels ng/ml**	***P***
Clinical characteristic*	Age at diagnosis of melanoma (years)	<55	40	22.5(16.8- 33.4)	0.441
		≥55	41	19.7(16.4- 24)	
	Gender	Male	43	19.5(13.8- 26)	0.477
		Female	38	22.8(17.8- 32.6)	
	BMI (kg/m^2^)	Normal weight	24	22.9(19.2- 31.6)	0.063
		Overweight	24	19.6(15.3- 26.3)	
		Obese	15	15.7(11.4- 25.4)	
	Fitzpatrick skin type	Dark	37	22.9(19.4- 32.6)	**0.005**
		Fair	44	18(15.5- 24.4)	
	Hair color	Dark	53	21.6(16.8- 26.6)	0.912
		Light	23	22.4(16.4- 30.6)	
	Eye color	Dark	38	20.5(13.2- 26.2)	0.777
		Light	37	22.2(17.3- 32.5)	
	Actinic damage	No	62	22(16.6- 29.3)	0.161
		Yes	16	18.5(16.3- 32.6)	
	Nevus Number	<50	34	21.2(13.7- 29.9)	0.266
		51-99	17	20.2 (17.8- 24.3)	
		>100	12	26.05 (19–36.6)	
**Habits of solar exposure and photoprotection**	Solar exposure before 10 years old	None/Mild	19	16.8(12–27.4)	**0.019**
		Severe/Moderate	59	22.5(17.3- 30.6)	
	Solar exposure 10–18 years old	None/Mild	20	17.8(14.5- 22.5)	0.08
		Severe/Moderate	57	22.9(17.3- 32.6)	
	Solar exposure over 18 years old	None/Mild	11	17.7(16.4- 23.9)	0.267
		Severe/Moderate	29	22.6(16.8- 30.2)	
	Photoprotection before 10 years old	Usually	25	22.6(17.3- 27.4)	0.569
		Never	47	20.8(15.7- 31.1)	
	Photoprotection 10–18 years old	Usually	16	22.6(17.4- 27)	0.672
		Never	33	21.8(16.6- 32.5)	
	Photoprotection over 18 years old	Usually	35	19.7(16.8- 27.4)	0.824
		Never	36	21.7(16–31.8)	
Melanoma characteristic*	Number of Melanoma	SPM	62	20.5(16.4- 27.4)	0.992
		MPM	19	21.6(16.8- 31.1)	
	Breslow thickness	<= 0.75	31	19(13.8- 29.3)	0.544
		0.75-1	15	22.2(17.8- 26)	
		> = 1	22	23.1(16.6- 31.1)	

The medians (25^th^ - 75^th^ percentiles) of 25-hydroxyvitamin D3 levels were reported. *P-values* were calculated by U Mann Whitney or Wilcoxon test.

*Not all the patients have complete data.

**Figure 1:**
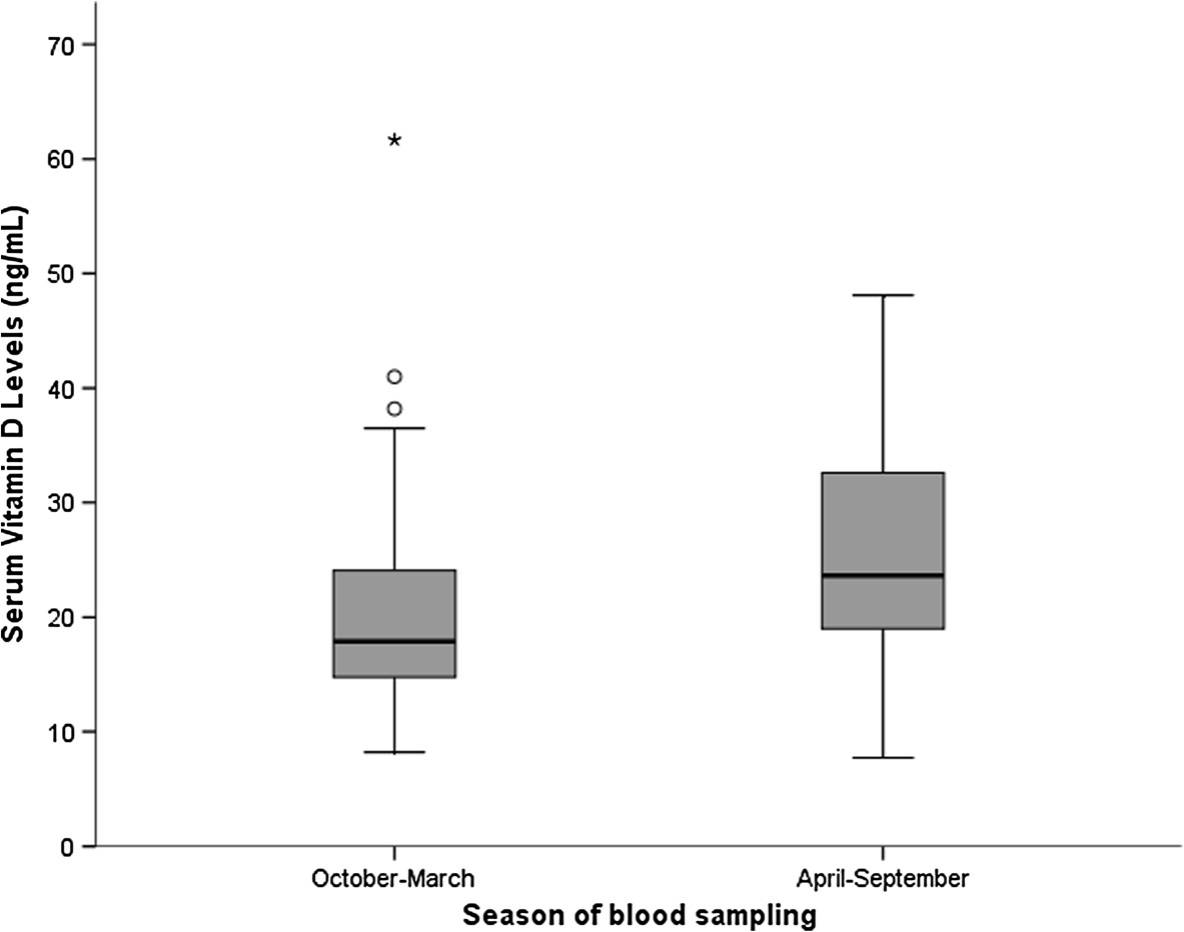
**Variation in serum 25-hydroxyvitamin D3 levels by season of blood sampling.** The circles represent high 25-hydroxyvitamin D3 values (38.2 ng/ml and 41 ng/ml) and the asterisk the highest 25-hydroxyvitamin D3 value 61.7 ng/ml.

Serum 25-hydroxyvitamin D3 levels varied with the season of venepuncture (Figure 1). The median level of 25-hydroxyvitamin D3 in April to September was 23.6 ng/ml, exceeding the average of the 25-hydroxyvitamin D3 levels in October to March of 17.9 ng/ml (*P* = 0.017).

It is known that BMI influences 25-hydroxyvitamin D3 levels. The 25-hydroxyvitamin D3 levels were shown to be lower (median 15.7 ng/ml) in obese patients with higher BMI (BMI ≥ 30) than in overweight patients with BMI between 25–29.9 (median 19.6 ng/ml) and in normal weight patients with BMI ≤ 24.9 (median 22.9 ng/ml) (*P* = 0.06).

Patients with phototype I/II had lower levels of 25-hydroxyvitamin D3 (median 18 ng/ml) than patients with phototype III/IV (median 22.9 ng/ml) (*P* = 0.005) in univariate analysis. Those patients who reported moderate/severe solar exposure before 10 years of age (≥ 50 h/y) had higher levels of 25-hydroxyvitamin D3 (median 22.5 ng/ml) than those with none/mild solar exposure (< 50 h/y) (median 16.8 ng/ml) (*P* = 0.019).

A higher percentage of patients with fair skin had none/mild solar exposure (<50 h/y) than dark-skinned patients in age groups <10 years (35% vs 12%; *P* = 0.06) (unpublished observations). The use of sunscreen was reported in 70% of patients. Skin type was also related with sunscreen: 62% of patients with light skin phototype usually used sunscreen compared with 29% in patients with darker skin phototype (*P =* 0.023) (unpublished observations).

As regards nevus number, patients with >100 showed higher 25-hydroxyvitamin D3 levels (26.3 ng/ml) than patients with 51–99 (20.2 ng/ml) or <50 nevi (21.2 ng/ml) but no significant differences were found among the three groups (*P* = 0.266).

Most of our patients (74%) had melanomas with Breslow thickness ≤ 2 mm (range from 0.2 to 7.5 mm).

Breslow was analyzed both as continuous and categorical variable. No statistically significant differences were found between Breslow thickness and 25-hydroxyvitamin D3 levels (r2 = 0.009, *P* = 0.42). The same result was found when examining the association between Vitamin D levels and Breslow as a categorical variable (*P* = 0.544).

No associations in levels of 25-hydroxyvitamin D3 were detected for actinic damage (*P =* 0.161), eye color (*P =* 0.777), hair color (*P =* 0.912), photoprotection habits before 10 (*P =* 0.569), between 10–18 (*P* = 0.672), over 18 years old (*P* = 0.824) and solar exposure between 10–18 (*P* = 0.08), over 18 years old (*P* = 0.267) and number of melanomas (*P =* 0.992) (Table 1).

25-hydroxyvitamin D3 level was also evaluated as a dichotomous variable. Two categories were defined: appropriate levels >25 ng/ml and insufficient levels ≤25 ng/ml. The cut off at 25 ng/ml was chosen as it is the range where the parathyroid hormone has its maximum inhibition [34]. Using this definition, 32% of patients had adequate levels (>25 ng/ml) and 68% had insufficient levels (≤25 ng/ml) of 25-hydroxyvitamin D3 (Table 2).

**Table 2:** Association between clinical characteristics and suboptimal 25-hydroxyvitamin D3 levels at time of diagnosis in melanoma patients

	Variables*		**VitD level sufficient >25 ng/mL n = 26(32%)**	**VitD level insufficient ≤25 ng/mL n = 55(68%)**	**OR (95%IC)**	***P***
**Clinical characteristic**	Age at diagnosis of melanoma**		54 (36–65)	55 (37–68)	0.99 (0.96- 1.01)	0.553
	Gender	Female	15 (57.7%)	23 (41.8%)	1	
		Male	11 (42.3%)	32 (58.2%)	1.9 (0.74- 4.88)	0.184
	BMI basal (kg/m2)	Normal weight	9 (47.4%)	15 (34.1%)	1	
		Overweight	6 (31.6%)	18 (40.9%)	0.56 (0.16- 1.92)	0.353
		Obese	4 (21.1%)	11 (25%)	0.61 (0.16- 2.49)	0.487
	Season blood sampling	Spring- Summer	18 (69.2%)	20 (38.5%)	1	
		Autumn- Winter	8 (30.8%)	32 (61.5%)	3.6 (1.32- 9.81)	**0.012**
	Fitzpatrick skin type	Dark	16 (61.5%)	21 (38.2%)	1	
		Fair	10 (38.5%)	34 (61.8%)	2.59 (0.99- 6.76)	**0.052**
	Hair color	Dark	8 (30.8%)	32 (61.5%)	1	
		Light	8 (32%)	15 (29.4%)	0.89 (0.32- 2.49)	0.818
	Eye color	Dark	11 (44%)	27 (54%)	1	
		Light	14 (56%)	23 (46%)	0.67 (0.26- 1.76)	0.415
	Actinic damage	No	21 (80.8%)	41 (78.8%)	1	
		Yes	5 (19.2%)	11 (21.2%)	0.89 (0.27- 2.89)	0.843
	Nevus Number	<50	12 (54.5%)	22 (53.7%)	1	
		≥50	10 (45.5%)	19 (46.3%)	0.97 (0.34- 2.73)	0.946
**Habits of solar exposure and photoprotection**	Solar exposure before 10 years old	Severe/Moderate	21 (80.8%)	38 (73.1%)	1	
		None/Mild	5 (19.2%)	14 (26.9%)	1.55 (0.49- 4.9)	0.458
	Solar exposure 10–18 years old	Severe/Moderate	23 (88.5%)	34 (66.7%)	1	
		None/Mild	3 (11.5%)	17(33.3%)	3.83 (1.01- 14.5)	**0.049**
	Solar exposure > 18 years old	Severe/Moderate	11 (91.7%)	18 (64.3%)	1	
		None/Mild	1 (8.3%)	10 (35.7%)	6.11 (0.68- 54.5)	0.105
	Photoprotection before 10 years old	Never	17 (68%)	30 (63.8%)	1	
		Usually	8 (32%)	17 (36.2%)	1.20 (0.43- 3.37).33)	0.724
	Photoprotection 10–18 years old	Never	14 (73.3%)	19 (63.3%)	1	
		Usually	5 (26,3%)	11 (36.7%)	1.62 (0.46- 5.73)	0.453
	Photoprotection over 18 years old	Never	15 (60%)	21 (45.7%)	1	
		Usually	10 (40%)	25 (54.3%)	1.79 (0.66- 4.80)	0.250
**Melanoma characteristic**	Number of Melanoma	SPM	24 (92.3%)	49 (89.1%)	1	
		MPM	2 (7.7%)	6 (10.9%)	0.68 (0.13- 3.63)	0.652
	Breslow thickness^+^	<= 0.75	22 (46.8%)	9 (42.9%)	1	
		0.75-1	11 (23.4%)	4 (19.0%)	0.89 (0.22- 3.54)	0.867
		> = 1	14 (29.8%)	8 (38.1)	1.4 (0.43- 4.48)	0.574
	Breslow thickness^+^**		0.8 (0.52- 1.3)	0.8 (0.52- 1.2)	1.1 (0.78- 1.54)	0.583

VitD = 25-hydroxyvitamin D3 *Not all the patients have complete data ^+^Breslow thickness was analized as categorical and continuos variable **The medians (25^th^ - 75^th^ percentiles) of 25-hydroxyvitamin D3 levels were reported for age at diagnosis of melanoma and breslow thickness.

The relationship between 25-hydroxyvitamin D3 levels, as a dichotomous variable, and clinical characteristics was showed in Table 2. The group with insufficient 25-hydroxyvitamin D3 level (≤25 ng/ml) differed from those with appropriate 25-hydroxyvitamin D3 (>25 ng/ml) in season of blood sampling (*P* = 0.012), solar exposure between 10–18 years (*P* = 0.049) and skin type (*P* = 0.05) (Table 2). Since patient recollection of solar exposure may be subject to response and recall bias, solar exposure variable was not included in multivariate analysis.

After multivariate logistic regression analysis, season of sampling remained the only predictors of 25-hydroxyvitamin D3 levels, independent of gender or skin type (Table 3).

**Table 3:** Multivariate models for prediction of presence of low levels of 25-Hydroxyvitamin D3

**N Model**	**Variables**	**OR (95%CI)**	***P***
1	Season blood sampling (October- March)	3.25 (1.17-9.03)	0.024
	Skin phototype (Fair)	2.21 (0.81- 6.04)	0.123
2	Season blood sampling (October- March)	3.3 (1.17- 9.27)	0.024
	Skin phototype (Fair)	2.33 (0.84- 6.46)	0.105
	Gender (Male)	1.88 (0.68- 5.21)	0.227

Model 1. Estimation of presence of low levels of 25-Hydroxyvitamin D3 by season blood sampling adjusted by skin phototype.

Model 2. Estimation of presence of low levels of 25-Hydroxyvitamin D3 by season blood sampling adjusted by skin phototype and gender.

Multivariate logistic regression models for estimation of presence of low levels of 25-Hydroxyvitamin D3 suggest that season blood sampling in autumn-winter (October-March) months is the only predictor of low 25-Hydroxyvitamin D3, independently from skin phototype and gender.

### *VDR* polymorphisms

Clinical characteristics, habits of solar exposure and melanoma data of patients are resumed in Table 4. The median age at melanoma diagnosis was 45 years (33–59.8), considering all patients. Significant differences between cases with <50 nevi and cases with >100 nevi were found such as age at melanoma diagnosis (*P* < 0.001) and number of melanomas (*P* < 0.001) (Table 4). Patients with multiple nevi were younger (median 39 years) and have more multiple melanomas (55%) than patients with few nevi (median 49 years; 20% with melanoma multiple).

**Table 4:** Clinical characteristics of patients with low (<50) and high (>100) nevus number

	Variables*		**Cases <50(%) n = 150**	**Cases >100(%) n = 113**	***P***
**Clinical characteristic**	Age at melanoma diagnosis*		49(34–64)	39(32–50)	<0.001
	Gender	Male	68(45.3)	51(45.1)	1.000
		Female	82(54.7)	62(54.9)	
	Fitzpatrick skin type	Dark	80(58.4)	43(45.7)	0.061
		Fair	57(41.6)	51(54.3)	
	Hair colour	Dark	107(77.5)	71(75.5)	0.723
		Light	31(22.5)	23(24.5)	
	Eye colour	Dark	90(66.2)	60(64.5)	0.795
		Light	46(33.8)	33(35.5)	
	Actinic Damage	No	99(85.3)	64(85.3)	0.988
		Yes	17(14.7)	11(14.7)	
**Habits of solar exposure**	Solar exposure before 10 years old	None/Mild	53(41.4)	28(29.5)	0.067
		Severe/Moderate	75(58.6)	67(70.5)	
	Solar exposure 10–18 years old	None/Mild	30(23.8)	26(27.7)	0.517
		Severe/Moderate	96(76.2)	68(72.3)	
	Solar exposure over 18 years old	None/Mild	26(20.6)	24(25.8)	0.367
		Severe/Moderate	100(79.4)	69(74.2)	
**Melanoma characteristic**	Number of melanoma	SPM	120(80)	50(44.2)	<0.001
		MPM	30 (20)	63(55.8)	

*Not all the patients have complete phenotype data.**The median (25^th^ - 75^th^ percentile) of age at diagnosis of melanoma was reported.

A total of 10 out of 11 SNPs were successfully genotyped in 263 melanoma patients. SNP rs739837 (*Bgl*I) failed genotype analysis. All the 10 SNPs followed HWE in the reference group (cases with <50) (Table 4). Genotyping quality was confirmed with 100% concordance between duplicates.

Differences in genotype distributions between cases with <50 nevi and cases with >100 nevi were significant for 3 *VDR* SNPs: rs2189480 (OR = 1.80, 95%IC (1.17-2.76); *P*_additive_ = 0.006), rs2239179 (OR = 0.67, 95%IC (0.46-0.99); *P*_additive_ = 0.044) and rs7975128 (OR = 0.51, 95%IC (0.35-0.76); *P*_additive_ = 0.0005), adjusted for age and gender. An additional SNP rs3782905, significant under recessive model (OR =0.21, 95%IC (0.07-0.66); *P*_recessive_ = 0.0025), was not further considered as this effect is likely due to small number of individuals homozygous for this rare allele with high nevus number. Use of the Bonferroni test supported a significant association with nevus number only for rs7975128. Although SNP rs2239179 was no longer significant (*P*_additive_ = 0.06), adjustment for number of melanomas, as confounding variable, did not result in substantial OR variation for all three SNPs (Table 5).

**Table 5:** **Association between*VDR*polymorphisms and nevus number**

	**SNP**	Position*	**Change**	**MAF%**	**HWE*P^***	**Genotype**	**Cases <50(%)**	**Cases >100(%)**	**OR (95%CI)**^**a**^	***P***_***additive***_^***a***^	**OR (95%CI)**^**b**^	***P***_***additive***_^***b***^
**1**	rs7136534	48294626	C/T	0.18	0.400	C/C	101 (68.7)	70 (65.4)	1.00			
						T/C	40 (27.2)	33 (30.8)	1.15 (0.65- 2.02)			
						T/T	6 (4.1)	4 (3.7)	0.78 (0.21- 2.94)			
						additive			1.03 (0.65- 1.62)	0.903	1.18 (0.73- 1.92)	0.504
**2**	rs11574027	48287373	G/T	0.10	1.000	G/G	119 (82.1%)	86 (78.9%)	1.00			
						T/G	25 (17.2%)	23 (21.1%)	1.35 (0.71-2.57)			
						T/T	1 (0.7%)	0 (0%)	NA			
						additive			1.19 (0.64- 2.21)	0.580	1.30 (0.67- 2.52)	0.431
**3**	rs11168287	48285414	G/A	0.44	0.869	G/G	43 (29.3)	36 (33.0)	1.00			
						A/G	72 (49.0)	57 (52.3)	0.99 (0.55- 1.76)			
						A/A	32 (21.8)	16 (14.7)	0.58 (0.27- 1.24)			
						additive			0.79 (0.55- 1.14)	0.212	0.83 (0.56- 1.23)	0.352
**4**	rs2238136	48277713	G/A	0.24	0.349	G/G	86 (60.1)	52 (52.5)	1.00			
						A/G	47 (32.9)	44 (44.4)	1.61 (0.93- 2.79)			
						A/A	10 (7.0)	3 (3.0)	0.47 (0.12- 1.82)			
						additive			1.11 (0.72- 1.72)	0.636	1.05 (0.66- 1.67)	0.845
**5**	rs3782905	48266167	C/G	0.36	0.483	C/C	52 (35.9)	42 (38.5)	1.00			
						C/G	74 (51.0)	63 (57.8)	1.07 (0.62- 1.83)			
						G/G	19 (13.1)	4 (3.7)	0.22 (0.07- 0.71)			
						additive			0.70 (0.46- 1.06)	0.134	0.69 (0.44- 1.08)	0.099
**6**	rs2189480	48263828	G/T	0.35	0.238	G/G **			0.21 (0.07- 0.66)			
						G/G	65 (45.5)	30 (28.6)	1.00			
						T/G	68 (47.6)	63 (60.0)	1.92 (1.09- 3.39)			
						T/T	10 (7.0)	12 (11.4)	2.97 (1.12- 7.87)			
						additive			1.80 (1.17- 2.76)	**0.006**	1.66 (1.05- 2.60)	**0.027**
**7**	rs2239179	48257766	A/G	0.44	0.192	A/A	35 (23.5)	36 (33.3)	1.00			
						A/G	83 (55.7)	58 (53.7)	0.70 (0.39- 1.26)			
						G/G	31 (20.8)	14 (13.0)	0.44 (0.20- 0.99)			
						additive			0.67 (0.46- 0.99)	**0.044**	0.67 (0.44- 1.02)	**0.060**
**8**	rs11574077	48252927	T/C	0.05	0.463	T/T	129(87.8)	104 (93.7)	1.00			
						T/C	17 (11.6)	7 (6.3)	0.54 (0.21- 1.38)			
						C/C	1 (0.7)	0 (0.0)	NA			
						additive			0.52 (0.21- 1.27)	0.135	0.45 (0.17- 1.18)	0.088
**9**	rs11168267	48251542	C/T	0.07	1.000	C/C	128(85.9)	98 (88.3)	1.00			
						T/C	21 (14.1)	12 (10.8)	0.68 (0.32- 1.48)			
						T/T	0 (0.0)	1 (0.9)	NA			
						additive			0.84 (0.41- 1.72)	0.626	0.84 (0.38- 1.84)	0.656
**10**	rs7975128	48245828	G/A	0.33	0.385	G/G	56 (38.6)	63 (58.9)	1.00			
						A/G	64 (44.1)	35 (32.7)	0.47 (0.27- 0.82)			
						A/A	25 (17.2)	9 (8.4)	0.29 (0.12- 0.69)			
						additive			0.51 (0.35- 0.76)	**0.0005**	0.58 (0.38- 0.87)	**0.007**

MAF minor allele frequency.

*SNP position from NCBI; ^ HWE Hardy –Weinberg Equilibrium for cases with low number of nevi (cases <50) as reference group; a = adjusted for age and gender; b = adjusted for age. gender and number of melanoma.

** recessive model for SNP rs3782905.

Sub-group analysis revealed that rs7975128 was associated with decreased risk of multiple primary melanoma (OR = 0.64 95%CI (0.43-0.94); *P*_additive_ = 0.021) and with light eye color (OR = 1.48 95%CI (1.00-2.19); *P*_additive_ = 0.049).

## Discussion

Our work is the first study to describe 25-hydroxyvitamin D3 insufficiency in melanoma patients from Spain. Furthermore, the association between *VDR* SNPs and nevus number, an established risk factor for melanoma, was also evaluated.

The optimal level of 25-hydroxyvitamin D3 remains contentious^4^. Several meta-analyses support the evidence that low 25-hydroxyvitamin D3 serum level, which is highly prevalent worldwide, is a risk factor for many chronic diseases including cancer [35]. The minimum desirable serum level of 25-hydroxyvitamin D3 has been suggested to be between 20 ng/ml and 30 ng/ml [36]. In 2011 The IOM committee reported guidelines stating that a level of 25-hydroxyvitamin D3 >20 ng/ml is needed for good bone and general health for practically all individuals [37]. However, the IOM report did not consider clinical and demographic variables (ie: race/ethnicity, adiposity, body composition, sun exposure, etc.) by which 25-hydroxyvitamin D3 levels are notoriously affected. Recently, a meta-analysis involving 59 231 healthy subjects from eleven prospective cohort studies suggested that the optimal 25-hydroxyvitamin D3 concentrations of between 30 ng/ml and 40 ng/ml (75 nmol/L and 100 nmol/L) are needed to reduce mortality [38]. The majority of authors site the cut off as <30 ng/ml as this level is reported to correlate with leveling off of the parathyroid hormone (PTH) level. The level chosen as cut off varied in studies in European populations [39]. In our study a conservative level of ≤25 ng/ml as 25-hydroxyvitamin D3 insufficiency was chosen as reported in a previous study in melanoma patients [5]. Using this definition, most of our patients (68%) at the time of melanoma diagnosis had suboptimal levels of 25-hydroxyvitamin D3. Newton-Bishop *et al.*[5] determined the levels of 25-hydroxyvitamin D3 in 1132 patients with melanoma from the North of England (Leeds), where insufficient levels of 25-hydroxyvitamin D3 was found in 64% of patients, a proportion comparable to our study. In the same study, 25-hydroxyvitamin D3 levels were associated with Breslow thickness. In contrast to the UK study, we did not found any association between 25-hydroxyvitamin D3 and Breslow thickness as the majority of our melanoma patients has Breslow thickness <2 mm.

The prevalence of insufficiency in Spain, a Mediterranean country with many hours of sunshine and where tanning is considered indicative of a healthy and attractive status, was therefore unexpected. 25-hydroxyvitamin D3 levels in general Spanish population have been reported to be insufficiency by studies tended to be small and in different sub-groups of the population [40-42]. The largest study analyzed 25-hydroxyvitamin D3 level in 1262 individuals from Spain and reported 25-hydroxyvitamin D3 insufficiency in one-third (33%) of the population [43].

Consistent with previous finding [5], there was seasonal variation in serum levels of 25-hydroxyvitamin D3 where 25-hydroxyvitamin D3 levels were lower in autumn-winter months.

The role of solar exposure in melanoma is complex as, on the one hand, it is a strong risk factor for melanoma, and, on the other, lack of solar exposure leads to 25-hydroxyvitamin D3 deficiency. It has been recently reported that regular weekend solar exposure is protective against melanoma [44] and is associated with increased 25-hydroxyvitamin D3 levels [45]. We have observed a correlation between less solar exposure during adolescence and insufficient 25-hydroxyvitamin D3 levels. However this result has to be interpreted with caution as self- reported past solar exposure is subject to recall bias.

In our study, 25-hydroxyvitamin D3 levels at the time of diagnosis were lower in fair-skinned patients than darker ones. Although this relationship was no longer statistically significant when corrected for season, this may reflect low statistical power. However, three other studies have in fact also reported that within white-skinned populations, fair skinned people have lower levels of 25-hydroxyvitamin D3 [45-47].

It was postulated that lower levels of 25-hydroxyvitamin D3 in individuals with fair skin reflect their habits of sun protection and in fact, this group of patients did report fewer hours of daily sun exposure and greater use of sunscreen than patients with darker skin type (data not shown). Our results support the hypothesis that increased photoprotection practices may contribute to increase 25-hydroxyvitamin D3 deficiency although the results are still contradictory [48-50].

The effects of 25-hydroxyvitamin D3 are mediated by *VDR* and SNPs in this gene are expected to influence the anti-proliferative activity of 25-hydroxyvitamin D3 in melanocytes. The potential role of functional *VDR* variants such as *Fok*I and *Bsm*I in melanoma risk has been assessed by meta-analysis studies [22,23]. In our study, significant associations have been detected between nevus number, the most potent phenotypic risk factor of melanoma and 3 out of 11 *VDR* SNPs. The SNP rs2189480 was associated with risk of having a high number of nevi whereas SNPs rs7975128 and rs2239179 with decreased risk of having a high number of nevi. All 3 SNPs are localized in the *VDR* coding region between SNPs *Fok*I and *Bsm*I and have been previously described in other pathologies [50-52]. After Bonferroni correction, only SNP rs7975128 remained significant.

When we analyzed the effect of *VDR* variant and melanoma susceptibility, we found that SNP rs7975218 was also associated with decreased risk of multiple primary melanomas. Recently, in a large case-only study, 8 *VDR* SNPs, 6 not previously studied, were associated with melanoma susceptibility [24]. As Orlow *et al.*[24] report, the present work confirmed the efficiency of the tagSNP method and showed that information about the genetic variability of *VDR* is still incomplete.

According to HapMap, rs7975218 is in complete LD (r2 = 1) with *VDR* functional variants *Bsm*I and *Taq*I (rs731236) which formed a block in tight LD, including also *Apa*I (rs7975232), located at the 3’UTR region of *VDR*. Consistent with previous findings in melanoma where, both *Taq*I and *Bsm*I have been shown to be protective against melanoma risk [21-23], SNP rs7975128 showed a protective tendency for high nevus number and number of melanoma.

By functional study, Carling *et al.*[53] have demonstrated that baT (haplotype of *Bsm*I/*Apa*I/*Taq*I) is associated with low VDR mRNA expression. The 3’UTR variants are thought to affect VDR mRNA stability [18] but the functionality of these variants is still unclear [22].

We also reported an association between SNP rs7975128 and light eye color, which, with pigmentary features such as light hair and fair skin, is considered a melanoma risk factor related to sun exposure. Although the significance of this association is not clear, the presence of this SNP among patients with light eye color could suggest the involvement of the *VDR* gene in melanoma predisposition among a subgroup of patients at risk of low 25-hydroxyvitamin D3. Relationship between *VDR* genetic variants and phenotype features among melanoma patients has been previously described [16].

The only report in a Spanish population found significant associations between *VDR* SNPs and clinical characteristics such as fair skin, absence of childhood sunburns and tumor in head-neck and trunk but not with melanoma risk, suggesting that *VDR* may modulate melanoma susceptibility [29].

Studies including only melanoma patients have been shown to be valid in identifying rare genetic variants associated with melanoma predisposition as rare variants have a higher prevalence among cases [24,54].

The limitations of our study should be considered. The small sample size of the two groups of patients may affect our results; however the proportion of patients with insufficient 25-hydroxyvitamin D3 is comparable to a previous study in a larger melanoma population [5]. Information regarding dietary and 25-hydroxyvitamin D3 supplement sources, conditions that may affect 25-hydroxyvitamin D3 levels, were not taken into account in the analysis as not available.

## Conclusions

Information on both serum 25-hydroxyvitamin D3 levels and *VDR* variants are required to understand the role of 25-hydroxyvitamin D3 in melanoma risk. High prevalence of sub-optimal levels of 25-hydroxyvitamin D3 was found among melanoma patients at diagnosis. The relationship between *VDR* SNP rs7975128 and, respectively, low nevus number and less multiple primary melanoma, suggests the involvement of VDR, and in turn of 25-hydroxyvitamin D3, in melanoma susceptibility.

Our results provide further evidence that 25-hydroxyvitamin D3 could be involved in melanoma etiology in a population from Barcelona, a Mediterranean area characterized by a sunny climate. However this finding needs to be validated in a larger sample.

## Competing interests

The authors declare that they have no competing interests.

## Authors’ contributions

ZO was involved in collecting the samples, genotyping analysis, acquisition of the data, association analysis, interpretation of the data and wrote the manuscript. LV was involved in acquisition of the 25-hydroxyvitamin D3 data, interpretation of the data and wrote the manuscript; CB, JAPB were involved in design of the study and collecting the samples; JMA performed 25-hydroxyvitamin D3 measurements; JR, NB, EG, MAP performed statistical analysis; CC, IK, JM were involved in patients recruitment; SP conceived the study, participated in its design and coordination, in interpretation of the data and drafting the manuscript. All authors contributed to critical revision of the manuscript and read and approved the final manuscript.

## Pre-publication history

The pre-publication history for this paper can be accessed here:

http://www.biomedcentral.com/1471-2350/14/26/prepub
